# A qualitative phenomenological study of student coping strategies in the VUCA era among first-year health students in Malang

**DOI:** 10.11604/pamj.2025.52.134.48767

**Published:** 2025-12-01

**Authors:** Dhian Kartikasari, Heni Dwi Windarwati, Viera Wardhani, Sri Andarini

**Affiliations:** 1 Department of Medicine, Faculty of Medicine, Brawijaya University and University of Malang, Malang, Jawa Timur, Indonesia,; 2 Department of Mental Health Nursing, Faculty of Health Sciences, Brawijaya University, Malang, Jawa Timur, Indonesia,; 3 Department of Family Medicine, Faculty of Medicine, Brawijaya University, Malang, Jawa Timur, Indonesia

**Keywords:** Coping strategies, academic stress, VUCA era, health students, social support

## Abstract

**Introduction:**

first-year students in health programs face major challenges in the volatility, uncertainty, complexity, and ambiguity (VUCA) era, marked by sudden academic changes, complex workloads, and future uncertainty. These pressures often trigger significant academic stress. While coping strategies are key in managing this stress, few studies have comprehensively examined the contextual factors influencing students´ coping choices. This study has identified the factors that shape students´ coping strategies in navigating academic stress during the VUCA era.

**Methods:**

this study employed a qualitative phenomenological design guided by the COREQ checklist. Data were collected through semi-structured, in-depth interviews conducted via Zoom with 21 purposively selected first-year health students at public universities in Malang, Indonesia. The interviews were transcribed verbatim and analyzed using the interpretative phenomenological analysis (IPA) framework to extract themes and personal meanings.

**Results:**

students´ coping strategies were influenced by three main factors: (1) sociodemographic conditions (such as monthly pocket money and region of origin); (2) motivation for choosing a major (amotivation, extrinsic, or intrinsic); and (3) forms of social support (emotional, instrumental, or informational). Students with strong emotional support and intrinsic motivation were more likely to use adaptive, problem-focused coping strategies, while those facing economic or cultural barriers often employed emotion-focused strategies.

**Conclusion:**

coping strategies among first-year health students in the VUCA era are shaped not only by stressors but also by internal and external contextual factors. These findings underscore the importance of early psychosocial interventions tailored to student characteristics to strengthen resilience and academic adaptation during periods of transition.

## Introduction

First-year students in health programs experience a significant transition period, during which they must adapt to a new learning environment that demands independence, strong academic competence, and effective social skills [1]. These challenges are further intensified under the VUCA framework, which characterizes a world of rapid change and systemic uncertainty [2]. In higher education, the VUCA era is reflected in sudden curriculum adjustments, unstable schedules and evaluation systems, and technological advances that are not yet fully integrated into learning processes, which have been associated with mild to severe depression in 68.5% of students and mild to severe anxiety in 54.4% of students [3].

Several studies have shown that healthy students show high vulnerability to academic stress, especially during the early years of their studies [4]. The pressure comes not only from academic load and performance demands, but also from external factors such as economic conditions, geographical distance from family, changes in the socio-cultural environment, and the demands of the role of new students in the health field. Stress that is not managed well can cause emotional disorders, decreased motivation to learn, problems in social relationships, and academic burnout [5]. This transition period increases the risk of psychological disorders, such as anxiety and depression, due to various stressors, including academic demands and changes in lifestyle [6]. A survey of 198 new students in Malang City revealed that 31.8% of respondents experienced cognitive disorders and 31.8% experienced emotional disorders. This indicates that first-year students are more vulnerable to mental disorders due to complex adaptation in a new academic environment [7]. Curriculum uncertainty and accelerated technological change also add to the burden of adaptation, often leading to emotional stress, anxiety, and mental health disorders.

In dealing with stress, students develop various coping strategies that aim to manage stress psychologically and behaviourally [8]. These coping strategies can generally be classified into two main approaches: problem-focused coping, which is oriented towards solving problems, and emotion-focused coping, which focuses on regulating emotions. However, the selection process and effectiveness of coping strategies are not consistent across individuals. Factors such as academic motivation, sociodemographic conditions (e.g., pocket money and region of origin), and social support from the surrounding environment are thought to significantly influence students' tendencies in choosing certain types of coping [9]. Although the role of coping strategies in dealing with academic stress has been widely discussed in the literature, there is still a gap in understanding the interaction and influence of various contextual factors on students' coping strategies, especially in the context of health higher education in the VUCA era [10]. A grounded theory approach is an appropriate method to explore these dynamics in depth based on students' actual experiences, as optimal coping strategies are key to stress management [11].

This study aims to identify and explain the factors that influence students' coping strategies in dealing with academic stress in the VUCA era, as well as to develop a conceptual model based on the empirical experiences of first-year health students [12]. In this study, the primary outcome was the type of coping strategy employed by first-year health students, classified as either problem-focused or emotion-focused. The main predictor variables included: (1) motivation for choosing a major (intrinsic, extrinsic, or amotivation); (2) social support (emotional, instrumental, informational); and (3) sociodemographic context (monthly pocket money, region of origin). Potential confounding factors that might influence coping behaviors include students´ prior mental health history, pre-university academic stress, and availability of personal resilience resources [13].

## Methods

**Study design and setting:** this study employed a cross-sectional qualitative phenomenological design, guided by the Consolidated Criteria for Reporting Qualitative Research (COREQ), to explore the lived experiences and coping strategies of first-year health students in adapting to academic stress during the VUCA era. This study used the interpretative phenomenological analysis (IPA) approach to extract deep, personal meanings from participants' narratives. The research was conducted remotely via Zoom meetings, targeting first-year health students enrolled at public universities in Malang, Indonesia. Data collection occurred between March and April 2025, allowing participation from geographically diverse regions. Each interview session lasted 40-60 minutes per participant and was conducted once, ensuring privacy and depth in responses.

**Figure 1:**
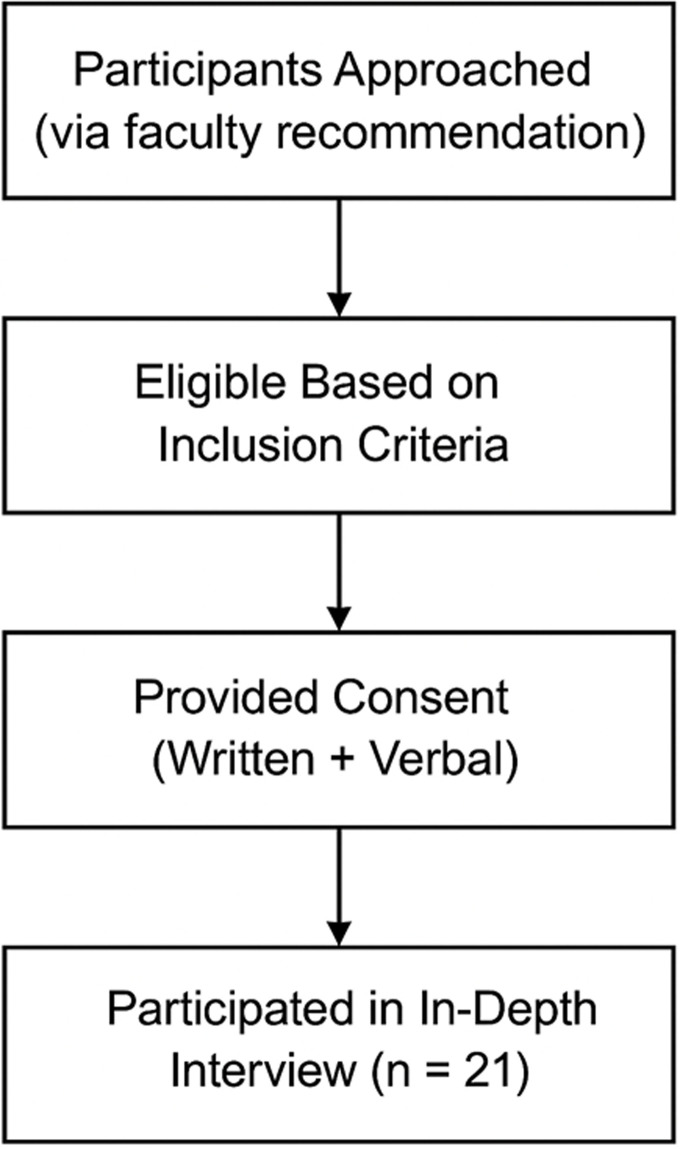
flow of participant inclusion in a qualitative phenomenological study of coping strategies among first-year health students in Malang, Indonesia, 2025 (n = 21)

**Participants:** a purposive sampling strategy was used to recruit participants who met the inclusion criteria of being currently enrolled as first-year university students and attending in-person lectures. Diversity was ensured by considering age, gender, semester level, high school major, and faculty affiliation. Students who declined participation were excluded. Data saturation was reached after the 21^st^interview, at which point no new themes emerged. The final sample, therefore, comprised 21 students, which aligns with methodological recommendations suggesting that 12-25 interviews are generally adequate for interpretative phenomenological analysis. To enhance variation, recruitment followed the principle of maximum diversity, ensuring representation across socioeconomic status, geographic origin, and health-related majors. Although no numeric quotas were predefined, the sample included students from both urban and rural areas, with monthly allowances ranging from low to high. The recruitment and enrolment process is summarized in Figure 1.

**Data collection:** data were gathered through semi-structured, in-depth interviews conducted individually via Zoom. Participants were invited based on recommendations from the heads of study programs. Written informed consent and sociodemographic data were collected before the interview began. Verbal consent for audio recording was obtained at the start of each session. Each interview followed a thematic interview guide developed around open-ended prompts such as: "what do you feel during the academic process at this university?" Field notes were taken during and after the interviews to capture nonverbal cues, emotional tone, and contextual behaviors. All interviews were conducted by the lead researcher, a doctoral candidate in Social Medicine at Brawijaya University. Clarification interviews were conducted when needed to ensure data accuracy. Interviews were transcribed verbatim and then cross-checked against recordings and field notes (Table 1).

**Table 1:** alignment of study themes, variables, and data sources in a qualitative phenomenological study of coping strategies among first-year health students in Malang, Indonesia, 2025 (n = 21)

Theme	Variable type	Interview focus question	Analysis approach
Sociodemographic context	Predictor	Tell me about your current living situation and financial condition	Coded for economic strain, adaptation
Motivation	Predictor	Why did you choose this study program?	Coded for intrinsic/extrinsic factors
Social support	Predictor	Who supports you when you're stressed?	Coded by type of support
Coping strategy	Outcome	What do you usually do when you're under academic pressure?	Classified as problem- or emotion-focused
Mental well-being history	Confounder (contextual)	Probed indirectly in follow-up if needed	Considered in interpretation only

**Data analysis:** as a qualitative study, no statistical tests were performed. Thematic analysis followed the IPA framework, enabling the researcher to interpret the psychological meaning behind student experiences. Transcripts were coded with participant identifiers, and analysis followed Smith´s multi-step process (reading, initial noting, theme development, and interpretative synthesis) (Table 2). Themes were interpreted iteratively from parts to the whole, triangulated with participant clarifications and nonverbal data, ensuring credibility, confirmability, dependability, and transferability. To ensure analytic transparency and conceptual rigor, the alignment between interview domains, participant quotes, sociodemographic data, and emergent themes is summarized in Table 3. This table clarifies how each data type contributed to theme development while distinguishing contextual variables from analytical units (Table 3).

**Table 2:** interpretative phenomenological analysis process applied in a qualitative study of coping strategies among first-year health students in Malang, Indonesia, 2025 (n = 21)

Analysis stages based on Smith's steps	Applications in this paper
Reading and re-reading	Researchers read it over and over again until they understood the position of important words
Initial noting	Analyze the contextual meaning of the words by looking for the actual meaning from various sources to obtain the true meaning of the word
Developing emergent themes	The researcher analyzed the sentences to formulate subthemes and important themes. Next, the researcher reflected on the sentences into subthemes and themes
Searching for the connection of cross-emergent themes	The researcher showed that the themes were interrelated and related to each other manually based on data coding
Moving on to the next cases	The analysis of subsequent participants up to the last participant is carried out according to the principles based on stages 1-4
Looking for patterns across cases	Researchers look for patterns that emerge in participants. From these patterns, researchers formulate them into themes
Taking interpretations to deeper levels	Researchers conducted a more in-depth and interpretive analysis to find out the original meaning

**Table 3:** alignment between interview domains, data sources, and thematic outcomes in coping strategy research among first-year health students in Malang, Indonesia, 2025 (n = 21)

Interview domain	Emergent theme	Supporting data type	Notes on use in analysis
Academic pressure and transitions	Stress perception in the VUCA context	Participant quotes	Thematic coding; quote frequency analysis
Motivation for major selection	Intrinsic vs. extrinsic/amotivation	Participant quotes + theme code	Used to classify coping orientation
Sources of social support	Emotional, instrumental, informative	Quotes + subtheme references	Clustered by type; not compared quantitatively
Financial and geographic context	Sociodemographic strain or advantage	Background info + participant story	Contextual framing; not outcome-driven
Coping approaches under stress	Problem-focused vs. emotion-focused	Quotes + behavior descriptors	Outcome variable derived from strategy typology
VUCA: volatility, uncertainty, complexity, and ambiguity			

**Measurement alignment across data sources:** to ensure analytic transparency, thematic development was grounded in predefined interview domains, and themes were inductively derived from participant narratives. Each emergent theme is supported by illustrative quotes and contextualized with relevant sociodemographic factors (e.g., age, pocket money, geographic origin) for depth, not for statistical comparison. Sociodemographic data functioned as a lens for interpretive framing rather than as analytical variables.

**Variables:** the following variables were identified in this study: 1) Outcome variable: type of coping strategy (problem-focused or emotion-focused); 2) predictor variables: academic motivation (intrinsic, extrinsic, amotivation), social support (emotional, instrumental, informational), sociodemographic factors (monthly pocket money, region of origin); 3) potential confounders: prior mental health history, personal resilience, or pre-university academic challenges. These variables were mapped to specific interview questions and coded thematically. Quantitative demographic characteristics were used descriptively (e.g., age, gender, finances), not for inferential analysis.

**Study size:** the final sample included 21 participants, with recruitment guided by the principle of theoretical saturation reached when no new themes emerged during subsequent interviews. Participants were purposively selected to ensure maximum variation across faculties and socioeconomic backgrounds, thereby enriching the thematic depth of the study. Given the cross-sectional qualitative design, the outcomes are inherently narrative and interpretative. As such, numerical outcome counts or event-based summaries are not applicable. Thematic findings emerged from participants´ lived experiences and are presented through a rigorous interpretive analysis.

**Bias and reflexivity:** to minimize selection bias, purposive sampling ensured diverse representation. Researcher reflexivity was maintained through field notes, peer debriefing, and continuous reflection on potential biases. Data were triangulated with nonverbal observations and follow-up clarifications to improve interpretive accuracy.

**Ethical considerations:** this study was conducted in accordance with the ethical principles outlined in the Declaration of Helsinki. Ethical approval was obtained from the Ethics Committee of the Faculty of Medicine, Brawijaya University, Malang (Approval No.: 356/EC/KEPK/02/2025, issued February 2025). All participants provided written informed consent and gave additional verbal consent for audio recording of the interviews. To ensure confidentiality, all data were anonymized using coded identifiers and securely stored. In line with institutional policy, data will be retained for five years.

## Results

This study does not employ adjusted estimates, as no quantitative confounder control was conducted due to its qualitative design. This qualitative study involved 21 informants who were first-year students from various study programs in the health science cluster. Most participants were 20 years old (66.7%) and female (71.4%), reflecting typical demographic patterns in health-related study programs. Their full demographic characteristics are presented in Table 4. The majority of informants were in the age range of 20 years (66.7%), followed by 19 years old at 28.5%, and only one informant was 21 years old (4.8%). This indicates that most informants were at the beginning of their undergraduate studies. Based on gender, there were 15 females (71.4%) and 6 males (28.6%), reflecting the dominance of female participation, which is also commonly found in the health study cluster in Indonesia (Table 4).

**Table 4:** sociodemographic characteristics of first-year health students in Malang, Indonesia, 2025

No	Demographic data	n (%)
1	**Age**	
	19	6 (28.5)
	20	14 (66.7)
	21	1 (4.8)
2	**Gender**	
	Man	6 (28.6)
	Woman	15 (71.4)
3	**Faculty of origin**	
	Nutritional science	2 (9.5)
	Pharmaceutical science	4 (19.1)
	Public health science	4 (19.1)
	Medical science	9 (42.9)
	Dentistry science	2 (9.5)
4	**Range of student pocket money per month**	
	<500,000	0
	500,000 - 1,000,000	7 (33.3)
	>1,000,000	14 (66.7)
5	**Region of origin/place of residence**	
	Java Island	16 (76.1)
	Outside Java Island	5 (23.9)

In terms of faculty origin, the informants came from five different study programs. A total of 9 participants (42.9%) were from the faculty of medicine, followed by public health and pharmaceutical sciences with 4 participants each (19.1%), and nutrition and dentistry with 2 participants each (9.5%). This demonstrates the diversity of academic backgrounds that enrich students´ experiences of academic stress in the VUCA era. Regarding economic conditions, most students had monthly allowances above IDR 1,000,000 (66.7%), while the remaining 33.3% received allowances between IDR 500,000 and IDR 1,000,000. There were no informants with allowances below IDR 500,000, but this range already illustrates significant variation in students´ financial capacity.

Geographically, 16 informants (76.1%) were from Java, while 5 (23.9%) came from outside Java, including Kalimantan, Sulawesi, and Nusa Tenggara. Students from outside Java often faced greater social and emotional adjustment challenges, such as language barriers, cultural differences, and limited support networks. One participant shared, *“moreover, sometimes they use Javanese, which I don´t understand”*, informant 20. In contrast, students from Java generally adapted more easily but still experienced emotional strain due to distance from family or close friends. These regional differences highlight how geographical and cultural contexts shape students´ adaptation experiences and influence their coping strategies.

Overall, variations in age, gender, study program, financial condition, and region of origin contribute to the diversity of students´ perspectives and experiences in dealing with academic stress and in forming coping strategies amidst the complexity of the VUCA era. This study revealed that the coping strategies used by first-year health students in managing academic stress in the VUCA era are influenced by several main contextual factors, namely sociodemographic conditions, motivation for choosing a major, and the type of social support received. The interaction of these three factors significantly shapes students' preferences for using certain coping strategies, either problem-focused or emotion-focused.

**Figure 2:**
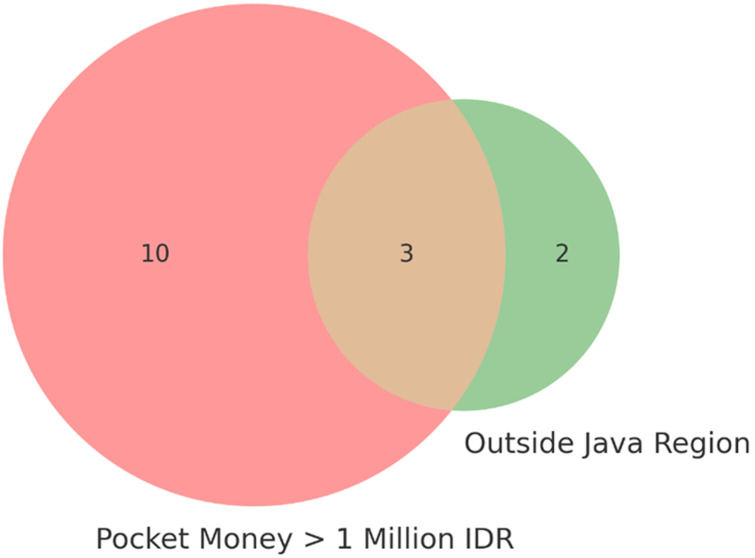
distribution of students by monthly pocket money and region of origin (Java vs. outside Java) among first-year health students

**Sociodemographic conditions:** students´ sociodemographic conditions, particularly the amount of monthly allowance and region of origin, play an important role in shaping their responses to academic pressure. Students with limited budgets (e.g., IDR 1,000,000 per month or less) face difficulties in meeting daily needs, including orientation expenses, stationery purchases, and daily consumption. Two informants stated: *“at college, I do everything alone, and it adds to my loneliness compared to when I was always with friends at home”*, informant 7; *“I was in a dilemma about going home for Eid because travel costs were high and I had just paid tuition”*, informant 2. Economic inequality and barriers to cultural adaptation influence students´ perceptions of academic pressure and determine the effectiveness of the coping strategies they employ. As shown in Figure 2, the distribution of students by monthly pocket money and region of origin illustrates variations in economic conditions and geographic backgrounds.

Economic inequality and barriers to cultural adaptation influence students' perceptions of academic pressure and determine the effectiveness of the coping strategies they employ. As shown in Figure 2, the distribution of students by monthly pocket money and region of origin illustrates variations in economic conditions and geographic backgrounds. Sociodemographic categories were shaped primarily by two aspects: the amount of monthly pocket money and the student´s region of origin. Among the 21 participants, 13 reported monthly allowances greater than IDR 1,000,000, and 5 came from outside Java. Most students with higher allowances were from Java, whereas only a few students from outside Java reported similar financial resources. These distributions are illustrated in Figure 2.

**Motivation for choosing a major:** unequal economic and cultural adaptation barriers affect students' perceptions of academic pressure and determine the effectiveness of their coping strategies. The reasons for choosing a study program are also considered as students' motivation in choosing a major, categorized into three types: amotivation, extrinsic motivation, and intrinsic motivation. Students who have a motivation tend to choose a major without a clear reason or because of coercion, which results in low emotional involvement in the learning process and an increased risk of confusion when facing academic pressure. In contrast, students with intrinsic motivation, characterized by internal drives such as an interest in knowledge or childhood aspirations, show more resilience in coping with stress.

**Figure 3:**
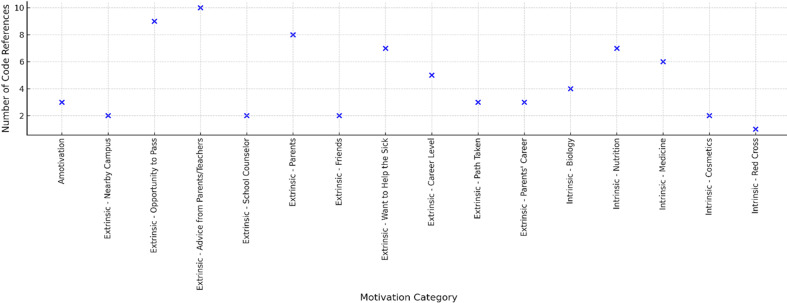
sources of motivation for major selection among first-year health students

*"I want to enter this major because I want to help sick people, it feels more meaningful”*, informant 12. Extrinsic motivations, such as the desire to obtain a good job, encouragement from parents, or strategies for getting into college, also emerge, although in some cases they do not provide the same psychological resilience as intrinsic motivation. *“My parents are in the health sector, so I also want to contribute like them”*, informant 9 (Figure 3).

Motivation for choosing a major was found to be predominantly extrinsic rather than intrinsic. Many students based their decisions on advice from parents, teachers, or friends (10 references), opportunities to pass university entrance selection (9 references), and direct encouragement from parents (8 references). These findings suggest that academic decisions were often shaped by social and pragmatic considerations rather than personal interest. Although intrinsic motivations such as an interest in medicine, biology, or nutrition were also reported, they appeared less frequently, indicating that internal drives were not the main factors influencing major selection. A small number of students expressed motivation, choosing their major without clear reasons, which may increase their vulnerability to academic stress. Figure 3 illustrates the dominant sources of motivation for major selection among students, highlighting the prevalence of extrinsic factors such as parental influence and employment prospects.

**Figure 4:**
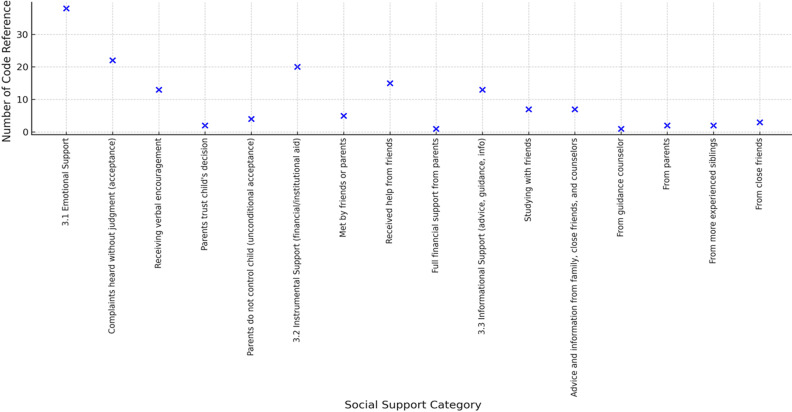
types of social support reported by first-year health students

**Social support:** social support falls into three main categories: emotional, instrumental, and informational support. Emotional support is crucial for students who are separated from their families or experiencing stress, and can be manifested through presence, empathy, and supportive communication. *“When I was with friends, I felt happier and less stressed, but in college I´m often alone”*, informant 7. *"My parents never demanded a certain GPA-what matters is that I stay healthy and responsible“*, informant 13. Instrumental support, such as financial assistance or group collaboration with friends, serves as a crucial buffer against stress: *“a friend helped me find books and materials when I was sick”*, informant 9. On the other hand, informative support in the form of advice from seniors, lecturers, or peers helps students make more focused academic decisions. *“My sister gave me information about the scholarship and how to apply for it, so I wasn´t confused anymore“*, informant 13 (Figure 4).

Social support was categorized into three forms: emotional, instrumental, and informative. Emotional support emerged as the most dominant factor in students´ coping strategies, reflected in frequent references to being listened to without judgment, receiving verbal encouragement, and having parents express trust in their decisions. This pattern indicates that students primarily sought acceptance and empathy when facing academic challenges. Instrumental support, such as financial assistance or practical help from friends, was mentioned less often, suggesting that non-financial resources were more influential in the coping process. Informative support, such as advice from seniors, lecturers, or peers, was present but relatively limited, indicating that while useful, it was not a major coping resource. As shown in Figure 4, emotional support emerged as the most frequently cited form of social support, followed by instrumental and informational support.

**Coping strategies:** coping strategies applied by students are divided into two main categories: problem-focused coping and emotion-focused coping. Students who apply the problem-focused coping approach tend to be proactive, designing learning strategies, making to-do lists, utilizing technology, and seeking support from friends. An informant stated, *"I work with my friends, so it's not too hard"*, informant 9. *"While listening to the lecturer's recording, I also finished other assignments"*, informant 1.

**Figure 5:**
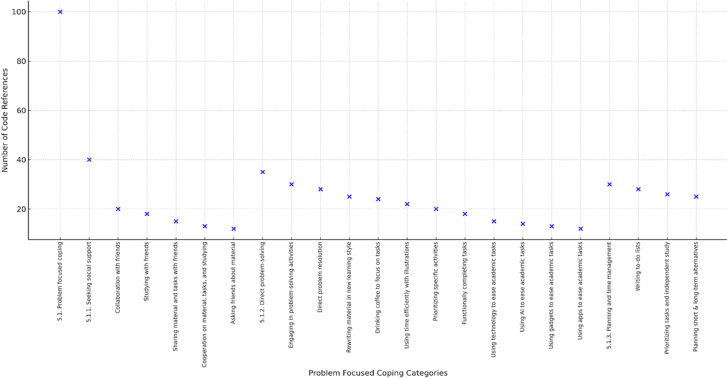
problem-focused coping strategies among first-year health students

In contrast, students who choose emotion-focused coping tend to manage their emotions without directly addressing the source of stress. These strategies include sleeping, praying, sharing feelings, or simply avoiding the task for a while: *“when I´m really stressed, I immediately perform ablution and pray"*, informant 2. *“I´m healing first, usually I just lie down in my boarding house”*, informant 6. This coping strategy is often applied when students feel that academic pressure is beyond their control or too much to handle directly. However, the choice of this strategy is also very much related to the capacity of support and resources available to students (Figure 5).

Students frequently relied on problem-focused coping strategies to manage academic and personal stressors. The most common approaches were direct problem solving and planning or time management, which were reflected in relatively high numbers of code references. Other strategies included reviewing material independently, multitasking during study sessions, and collaborating with peers to better understand assignments. Although seeking social support also appeared as part of this coping style, it was cited less often than direct problem-solving approaches. Overall, these findings suggest that students preferred practical and action-oriented responses over avoidance or purely emotional outlets. Figure 5 illustrates the range of problem-focused coping strategies reported by students, highlighting their preference for direct problem solving and time management.

**Figure 6:**
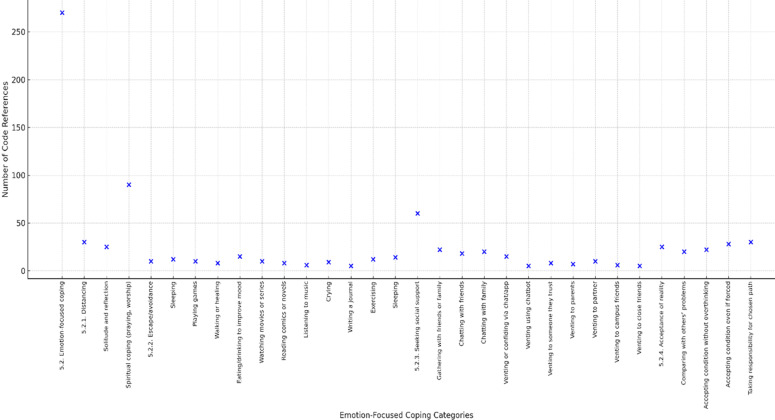
emotion-focused coping strategies among first-year health students

Students also relied on emotion-focused coping strategies, often turning to avoidance or social outlets rather than directly addressing stressors. Common approaches included shopping, playing with gadgets, and watching movies, which served as distractions to temporarily reduce negative feelings. Many participants also coped by confiding in friends or family, reflecting the importance of social sharing in emotional regulation. In contrast, strategies such as acceptance of change or personal reflection were less frequently reported, indicating that students tended to rely more on avoidance and external comfort than on introspection. As shown in Figure 6, emotion-focused coping strategies such as avoidance, distraction, and social sharing were also commonly used among students.

## Discussion

This study uses a qualitative phenomenological approach, especially with the COREQ checklist as a guide to identify factors that influence students' choice of coping strategies in dealing with academic stress in the VUCA era [14]. Based on in-depth interviews with 21 students, the data show that students tend to adopt two main types of coping strategies: problem-focused coping and emotion-focused coping. This finding is in line with the theory of Sutton *et al*. which explains that the coping process consists of two main dimensions: direct problem-focused coping and emotion-focused coping [15].

**Sociodemographic conditions:** students´ coping strategies are not formed in a vacuum, but are the result of a complex interaction between academic stressors, personal resources (motivation, values, cognitive abilities), and external resources (social support, access to information, and economic conditions) [16]. These factors are elements of contextual conditions that shape how individuals assess stressful situations and determine appropriate responses. For example, students from families with lower-middle economic status often face challenges not only in academic aspects, but also in financial demands. When they have to buy equipment for assignments or participate in certain academic activities that require costs, they face a dilemma between educational needs and financial limitations [17]. This exacerbates the psychological burden and poses a risk of reducing the effectiveness of coping. One informant stated, *“as students living independently, we often feel that expenses accumulate continuously”*, informant 8 (pocket money 1,000,000 per month), which indicates the existence of economic pressure related to academic stress.

Additionally, students with pocket money of more than one million showed better adaptability; they were able to get things that made their emotions more positive and neutralized stress. *"Later I will eat or drink coffee that can increase my energy or enthusiasm to do my assignments"*, informant 10 (pocket money 3,000,000 per month) [18].

Furthermore, the region of origin is divided into two categories, namely students from Java and students from outside Java. The majority of students come from Java, but there are several informants who come from outside the region, such as Kalimantan, Nusa Tenggara, and Sulawesi. Students from outside Java show greater adaptation challenges, especially in social and emotional aspects [19]. They feel distant from their families, have difficulty building relationships due to differences in communication styles or habits, and feel they do not have an environment that “connects”. These difficulties not only impact their daily lives but also weaken the social support they can access, further increasing feelings of alienation and psychological pressure [20]. This finding aligns with previous research indicating that regional and cultural distance can heighten acculturative stress and reduce academic adaptation. *“Especially if sometimes they use Javanese, which I don´t understand”*, informant 20, from outside Java.

Meanwhile, students from Java generally adapt faster, although they still experience emotional impacts due to losing relationships with old friends or not having time to go home. *"Well, in college, because I haven't found any really close friends, I'm alone"*, informant 9, from Java Island. *“Then I see friends who come home every week, if their house is close to Malang. It´s like I want to do that”*, informant 2, from Java Island. Students have the task of adjusting themselves based on their financial conditions and differences in geographical conditions. For example, avoiding snacks, limiting social activities, and refraining from going home for a full semester [19].

Although not explicitly stated as stressors, these pocket money conditions and geographical differences appear to be important backgrounds in the formation of perceptions of academic and social pressure [21]. An individual's stress response is determined by the appraisal process of a situation and the resources they have to deal with it. This process consists of two main stages, namely primary appraisal of how someone assesses a situation as dangerous or not, and secondary appraisal of the evaluation of one's abilities and available resources to deal with the situation. Sociodemographic factors such as economic status and social environmental conditions can influence both of these appraisal processes [22].

**Motivation:** furthermore, in the context of this study, students with substantial motivation and social support from family, peers, or the campus environment tend to perceive academic pressure as a challenge [23]. This assessment allows them to develop adaptive coping strategies, such as problem-focused coping, in which students actively try to complete tasks, find solutions through collaboration, or manage time more efficiently [24]. Moreover, students with low motivation, financial constraints, or those from remote areas tend to perceive pressure as a threat. This assessment culminates in the use of emotion-focused or even avoidance coping strategies, which do not directly resolve the problem and can lead to long-term distress [25].

Decisions that do not come from a personal consideration process make them more susceptible to stress when having to adapt to the rhythm and demands of study. *"Like I feel worried all the time. I'm sure the assignment is finished”* [26]. *“Will there be anything later? 3x"*, informant 11. Meanwhile, extrinsic motivation appears in the form of encouragement to get a decent job, promising job prospects, or because of the assumption that the opportunity to enter the major is big [27]. *"But my brother is a doctor, so my parents told him to become a doctor. It so happened that my parents told him to be a dentist"*, informant 18. Intrinsic motivation is reflected in students who choose a major because of their interest in the science [28]. This type of motivation is a strong source of psychological energy because it is directly related to personal goals and individual values [29]. *"Since school, I´ve liked biology and wanted to become a doctor"*, informant 11.

It is emphasized that motivation influences perceptions of threat and perceptions of the effectiveness of coping strategies [30]. Individuals who have strong goals are better able to survive stressful situations than those whose motivations are unclear or dependent on external factors. Students who understand why they are in the major will view obstacles as part of a valuable journey and understand the importance of persisting [31].

**Social support:** on the other hand, students who have strong social support in the form of emotional support tend to have a more optimistic perspective on academic challenges [32]. They tend to interpret stress as an integral component of personal development. This creates a sense of emotional safety and helps students feel accepted even when they are not at their best [33]. *"My family always listens and encourages me"*, informant 12. Meanwhile, instrumental support refers to support in the form of finance, energy, or physical presence; no need to worry about money problems, and one can focus on studying, visiting, or meeting with family when stressed [34]. This presence is not only symbolic but also a concrete form of assistance in completing academic burdens. *"Parents say 'don't think about the economy', that's their business"*, informant 2 [35]. Assistance in the financial aspect also provides space for students to survive amidst pressure, even in limitations. However, not all students have equal access to this type of support, depending on the financial conditions of each family [36].

Therefore, in the dynamic and uncertain context of higher education, such as the VUCA period, students' ability to accurately evaluate stress and access appropriate coping strategies greatly influences their academic success and psychological stability [37]. Therefore, an individualized and contextual support approach is essential in developing intervention programs in higher education environments, whether through counseling services, stress management skills training, or strengthening social support systems [38].

**Limitations:** this study has several limitations that should be acknowledged when interpreting the findings. First, the study employed a qualitative phenomenological approach with a relatively small, purposively selected sample of 21 students from health-related study programs in a specific geographic region (Malang, Indonesia). While this design allows for in-depth exploration of personal experiences, it limits the generalizability of the findings to broader student populations or other academic disciplines. Second, self-reported data through interviews may introduce recall bias or social desirability bias, as participants may have selectively shared experiences or framed their responses in socially acceptable ways. Although triangulation with field notes and clarifications with participants were used to enhance data credibility, these biases cannot be entirely ruled out.

Third, as all interviews were conducted online via Zoom, nonverbal cues were limited by screen-based observation, potentially affecting the richness of contextual interpretation. Furthermore, cultural and language differences between students from within and outside Java may have influenced the clarity of expression or understanding of questions, even though efforts were made to ensure clarity and rapport [39]. Lastly, the study's cross-sectional nature captured coping strategies at one point in time during early academic life. Coping behaviors are dynamic and may evolve as students gain experience or face different stressors. Therefore, longitudinal research would be valuable to track changes in coping over time. Future research may benefit from employing mixed-method or longitudinal designs to explore how coping strategies evolve throughout students´ academic journeys. Despite these limitations, the findings provide important insights into the contextual factors influencing student coping strategies and offer a foundation for developing more targeted psychosocial support programs in higher education.

## Conclusion

The model of students' coping strategies in the VUCA era shows that coping strategies are formed through complex interactions between academic pressure and students' internal and external resources. Factors such as motivation, social support, and sociodemographic conditions are important elements in shaping the type of coping strategies used. Students with strong intrinsic motivation and social support tend to use more adaptive problem-focused coping strategies. The implication of this finding is the importance of developing psychosocial interventions based on student characteristics to strengthen mental resilience and adaptive coping strategies from the beginning of the study period.

### What is known about this topic

- Academic stress is highly prevalent among first-year health students, especially during periods of major transition;
- Coping strategies are generally categorized as problem-focused or emotion-focused responses to stress;
- Sociodemographic factors and academic motivation are believed to influence stress perception and coping behaviors.

### What this study adds

- This study identifies how the interaction between motivation, social support, and financial/geographic background shapes coping strategy preferences in the VUCA era;
- It reveals that intrinsic motivation and emotional support are strong predictors of adaptive, problem-focused coping;
- The findings propose a conceptual model for psychosocial interventions tailored to student characteristics during early university life.

## References

[1] Pryjmachuk S, McWilliams C, Hannity B, Ellis J, Griffiths J (2019). Transitioning to university as a nursing student: Thematic analysis of written reflections. Nurse Educ Today.

[2] Mirza AA, Baig M, Beyari GM, Halawani MA, Mirza AA (2021). Depression and Anxiety Among Medical Students: A Brief Overview. Adv Med Educ Pract.

[3] Zhang J, Peng C, Chen C (2024). Mental health and academic performance of college students: Knowledge in the field of mental health, self-control, and learning in college. Acta Psychol (Amst).

[4] Wyatt TJ, Oswalt SB, Ochoa Y (2017). Mental Health and Academic Performance of First-Year College Students. International Journal of Higher Education.

[5] Zada S, Wang Y, Zada M, Gul F (2021). Effect of mental health problems on academic performance among university students in Pakistan. Int J Ment Health Promot.

[6] Hamaideh SH (2011). Stressors and Reactions to Stressors Among University Students. Int J Soc Psychiatry.

[7] Hadar LL, Ergas O, Alpert B, Ariav T (2020). Rethinking teacher education in a VUCA world: student teachers´ social-emotional competencies during the Covid-19 crisis. Eur J Teach Educ.

[8] Iorga M, Dondas C, Zugun-Eloae C (2018). Depressed as Freshmen, Stressed as Seniors: The Relationship between Depression, Perceived Stress and Academic Results among Medical Students. Behav Sci (Basel).

[9] Ryff CD (2014). Psychological well-being revisited: advances in the science and practice of eudaimonia. Psychother Psychosom.

[10] Olmos-Vega FM, Stalmeijer RE, Varpio L, Kahlke R (2022). A practical guide to reflexivity in qualitative research: AMEE Guide No. 149. Med Teach.

[11] Franconeri SL, Padilla LM, Shah P, Zacks JM, Hullman J (2021). The Science of Visual Data Communication: What Works. Psychol Sci Public Interest.

[12] Nyimbili F, Nyimbili L (2024). Types of Purposive Sampling Techniques with Their Examples and Application in Qualitative Research Studies. Br J Multidiscip Adv Stud.

[13] Heath J, Moran M, Dowrick A (2024). Examining qualitative cross-country comparative analysis in health: Reflective insights and methodological considerations. SSM Qual Res Health.

[14] McGrath C, Palmgren PJ, Liljedahl M (2018). Twelve tips for conducting qualitative research interviews. Med Teach.

[15] Sutton J, Austin Z (2015). Qualitative Research: Data Collection, Analysis, and Management. Can J Hosp Pharm.

[16] McMullin C (2023). Transcription and Qualitative Methods: Implications for Third Sector Research. Voluntas.

[17] Busetto L, Wick W, Gumbinger C (2020). How to use and assess qualitative research methods. Neurol Res Pract.

[18] Yao M, Kadetz PI, Sidibe AM, Wu Y, Li J, Lyu J (2021). Teachers' Perceptions of Student Mental Health in Eastern China: A Qualitative Study. Int J Environ Res Public Health.

[19] Kristiana IF, Karyanta NA, Simanjuntak E, Prihatsanti U, Ingarianti TM, Shohib M (2022). Social Support and Acculturative Stress of International Students. Int J Environ Res Public Health.

[20] Tuco R, Culajara KB (2024). Exploring the life and experiences of women in a VUCA environment. Am J Arts Human Sci.

[21] Allaili A, Dewi EI, Kurniyawan EH (2021). The relationship between culture shock and Self-Esteem of new students outside Java Island at University of Jember. Nurs Health Sci J.

[22] Olagunju NOD, Assumang NDK, Boansi NSO, Achumba NU, Olaiya NOP, Adesoga NTO (2024). Cultural adaptation and its impact on the academic success and well-being of international students in U.S. higher education. GSC Adv Res Rev.

[23] Lim WM (2024). What is qualitative research? An overview and guidelines. Australas Mark J (AMJ).

[24] Melese AK, Pedro A, Somhlaba NZ (2024). The direct effect of basic need services, and social support on positive mental health among institutionalized children: the mediating role of psychological capital. Curr Psychol.

[25] Vicary E, Kapadia D, Bee P, Bennion M, Brooks H (2025). The impact of social support on university students living with mental illness: a systematic review and narrative synthesis. J Ment Health.

[26] Al-Tameemi RA, Johnson C, Gitay R, Abdel-Salam AS, Al Hazaa K, BenSaid A (2023). Determinants of poor academic performance among undergraduate students-A systematic literature review. Int J Educ Res Open.

[27] Yano K, Endo S, Kimura S, Oishi K (2021). Effective coping strategies employed by university students in three sensitivity groups: a quantitative text analysis. Cogent Psychol.

[28] Polin MB, Lerik MDC, Benu JMY (2023). The optimism and Problem-Focused coping in students who work Part-Time in the city of Kupang. J Health Behav Sci.

[29] Mmari K, Mafuta E, Yu C, Pinandari A, Borges ALV, Maddaleno M (2024). Coping Strategies Among Adolescents During the COVID-19 Pandemic: A Cross-Cultural Exploration. J Adolesc Health.

[30] Saeed M, Ullah Z, Ahmad I (2020). A qualitative exploratory study of the factors causing academic stress in undergraduate students in Pakistan. Liberal Arts Soc Sci Int J.

[31] Moreno-Montero E, Del Mar Ferradás M, Freire C (2024). Personal Resources for Psychological Well-Being in University Students: The Roles of Psychological Capital and Coping Strategies. Eur J Investig Health Psychol Educ.

[32] Rodrigues R (2020). Legal and human rights issues of AI: Gaps, challenges and vulnerabilities. J Respons Technol.

[33] Munandar AA (2020). Majapahit and the Contemporary Kingdoms: Interactions and views. Bull Archaeol.

[34] von Philipsborn P, Stratil JM, Burns J, Busert LK, Pfadenhauer LM, Polus S (2019). Environmental interventions to reduce the consumption of sugar-sweetened beverages and their effects on health. Cochrane Database Syst Rev.

[35] Chen C, Zhu Y, Xiao F, Que M (2023). Academic Motivation and social support: Mediating and moderating the Life Satisfaction and Learning Burnout link. Psychol Res Behav Manag.

[36] Sarmiento LF, Lopes da Cunha P, Tabares S, Tafet G, Gouveia A (2024). Decision-making under stress: A psychological and neurobiological integrative model. Brain Behav Immun Health.

[37] Bandhu D, Mohan MM, Nittala NAP, Jadhav P, Bhadauria A, Saxena KK (2024). Theories of motivation: A comprehensive analysis of human behavior drivers. Acta Psychol (Amst).

[38] Xin T, Siponen M, Chen S (2021). Understanding the inward emotion-focused coping strategies of individual users in response to mobile malware threats. Behav Inform Technol.

[39] Khatri P, Duggal HK, Lim WM, Thomas A, Shiva A (2024). Student well-being in higher education: Scale development and validation with implications for management education. Int J Manag Educ.

